# Similar efficacy and safety of artemether-lumefantrine (Coartem^®^) in African infants and children with uncomplicated falciparum malaria across different body weight ranges

**DOI:** 10.1186/1475-2875-10-369

**Published:** 2011-12-16

**Authors:** Quique Bassat, Raquel González, Sónia Machevo, Alain Nahum, John Lyimo, Hamma Maiga, Andreas Mårtensson, Mahfudh Bashraheil, Peter Ouma, David Ubben, Verena Walter, Obiyo Nwaiwu, Chemtai Kipkeu, Gilbert Lefèvre, Bernhards Ogutu, Clara Menéndez

**Affiliations:** 1 Barcelona Centre for International Health Research (CRESIB), Hospital Clínic, Institut d'Investigacions Biomèdiques August Pi i Sunyer, Universitat de Barcelona, Rosselló 132, 4°, 08036 Barcelona, Spain; 2 Centro de Investigação em Saúde de Manhiça (CISM), Manhiça, Mozambique; 3 Centre de Recherche Entomologique de Cotonou, Cotonou, Benin; 4 Ifakara Health Research and Development Centre, Dar es Salaam, Tanzania; 5 Malaria Research and Training Center, University of Bamako, Bamako, Mali; 6 Division of Global Health, Dept of Public Health Sciences, Karolinska Institutet, Unit of Infectious Diseases, Dept of Medicine, Karolinska Institutet/Karolinska University Hospital, Stockholm, Sweden and Zanzibar Malaria Research Unit of the Karolinska Institutet, Zanzibar, Tanzania; 7 Kenya Medical Research Institute, Kilifi, Kenya; 8 Kenya Medical Research Institute/Center for Disease Control, Kisumu, Kenya; 9 Medicines for Malaria Venture (MMV), Geneva, Switzerland; 10 Novartis Pharma AG, Basel, Switzerland; 11 Novartis Pharma AG, Mushin, Lagos, Nigeria; 12 Novartis Pharma Services AG, Africa Re Centre, Hospital Road, Nairobi, Kenya; 13 Walter Reed Project, Kenya Medical Research Institute, Kisumu, Kenya

## Abstract

**Background:**

Artemisinin-based combination therapy, including artemether-lumefantrine (AL), is currently recommended for the treatment of uncomplicated *Plasmodium falciparum *malaria. The objectives of the current analysis were to compare the efficacy and safety of AL across different body weight ranges in African children, and to examine the age and body weight relationship in this population.

**Methods:**

Efficacy, safety and pharmacokinetic data from a randomized, investigator-blinded, multicentre trial of AL for treatment of acute uncomplicated *P. falciparum *malaria in infants and children in Africa were analysed according to body weight group.

**Results:**

The trial included 899 patients (intent-to-treat population 886). The modified intent-to-treat (ITT) population (n = 812) comprised 143 children 5 to < 10 kg, 334 children 10 to < 15 kg, 277 children 15 to < 25 kg, and 58 children 25 to < 35 kg. The 28-day PCR cure rate, the primary endpoint, was comparable across all four body weight groups (97.2%, 98.9%, 97.8% and 98.3%, respectively). There were no clinically relevant differences in safety or tolerability between body weight groups. In the three AL body weight dosing groups (5 to < 15 kg, 15 to < 25 kg and 25 to < 35 kg), 80% of patients were aged 10-50 months, 46-100 months and 90-147 months, respectively.

**Conclusion:**

Efficacy of AL in uncomplicated falciparum malaria is similar across body weight dosing groups as currently recommended in the label with no clinically relevant differences in safety or tolerability. AL dosing based on body weight remains advisable.

## Background

Growing resistance to conventional anti-malarial drugs and the associated resurgence in infection rates and malaria-related morbidity and mortality, particularly in sub-Saharan Africa [1], has led to a paradigm shift in treatment strategies. Since 2004, the World Health Organization (WHO) recommends treatment with artemisinin-based combination therapy (ACT) [2], and ACT has been adopted as first-line treatment for uncomplicated *Plasmodium falciparum *malaria in virtually all African countries. Artemether-lumefantrine (AL), the first fixed-dose ACT to be prequalified by the WHO, has consistently shown PCR-corrected cure rates > 95% against this species, with prompt resolution of parasitaemia and fever, rapid gametocyte clearance and good tolerance in populations of adults and children [3-9], even when administered unsupervised [3,9-11]. Changes in parasite clearance in south-east Asia associated with altered artemisinin sensitivity are not yet relevant in most malarious areas of the world, although some declining responsiveness of *P. falciparum *infections to ACT (including AL) from non-Asian areas have been reported [12]. While consumption of milk or food enhances lumefantrine absorption, adequate exposure is achieved with a standard African diet [13,14]. Due to the short half-life of artemether [15], AL is administered twice a day to ensure that infecting parasites are exposed to artemether above the minimum effective concentration [16] throughout most of their life cycle [17]. A three-day regimen of AL is used to cover two full parasite life asexual cycles [17] and thus maximize efficacy [18]. High levels of adherence to this regimen have been described when AL is employed in the community [19].

Since AL was first licensed in 1999, it has been recommended that the drug be dosed according to body weight ranges, i.e. 5 to < 15 kg, 15 to < 25 kg, 25 to < 35 kg and > 35 kg [20]. This raises the question of whether patients at the lower or upper end of a category could be over- or underexposed to the drug. It is specifically important to establish whether infants and children in each weight category vary in terms of drug exposure or outcomes (i.e. efficacy, safety and tolerability) in relation to other weight groups. In the current analysis, data from a randomized, multi-centre, investigator-blinded study of AL involving 899 children in five African countries were evaluated to compare the efficacy and safety of AL between patients in the three body weight ranges, and to examine the relationship between age and body weight in this population.

## Methods

### Study design

This was a randomized, investigator-blinded, multi-centre trial undertaken to compare the efficacy, safety and tolerability of AL using a six-dose regimen of the dispersible tablet formulation versus standard crushed tablets in the treatment of acute uncomplicated *P. falciparum *malaria in infants and children [21]. The study was conducted at eight health care facilities in Benin, Kenya, Mali, Mozambique and Tanzania/Zanzibar. Full details of the study design and conduct have been published previously [21]. This study has been registered at controlled-trials.gov as NCT00386763.

### Patients

The study population comprised infants and children with microscopically confirmed acute uncomplicated *P. falciparum *malaria. Key inclusion criteria were age ≤12 years, body weight ≥5 kg and < 35 kg, the presence of fever (≥37.5°C axillary or ≥38°C rectally) or a history of fever in the preceding 24 h, *P. falciparum *malaria (mono or mixed infection) with an asexual blood density ≥2,000/μL and < 200,000/μL, and the absence of severe signs of complicated malaria as defined by WHO [22]. Key exclusion criteria included haemoglobin < 5 g/dL, intake of anti-malarials other than chloroquine within the previous 2 weeks (or up to a month if the drug taken was AL), ongoing prophylaxis with drugs with anti-malarial activity such as cotrimoxazole medication, use of any drug known to influence cardiac function in the preceding 4 weeks, corrected QT interval (QTc) prolongation (males > 450 ms, females > 470 ms) or any condition known to prolong QTc, and any serious underlying disease.

### Treatment

Eligible patients were randomly allocated to either dispersible or crushed tablets on a 1:1 ratio within each of the three body weight dosing groups: 5 to < 15 kg, 15 to < 25 kg, or 25 to < 35 kg. AL was administered twice daily for 3 days (six doses in total) with dosage determined according to body weight: one tablet per dose for children 5 to < 15 kg, two tablets per dose for those 15 to < 25 kg, and three tablets per dose for those 25 to < 35 kg. One tablet contains 20 mg artemether and 120 mg lumefantrine. Dosages according to weight show important variability between the different weight groups, with the minimum and maximum levels of drug intake both occurring in the 5-15 kg group, in which artemether intake ranges from 1.33 to 4 mg/kg and lumefantrine intake ranges from 8 to 24 mg/kg for each dose administered. Variability in the other two weight groups is much lower.

Patients were hospitalized during the three-day treatment phase and followed on a weekly basis up until day 42 after initiation of treatment. Treatment was administered under supervision and consumption of some food or drink (e.g. breast milk, broth, or sweetened condensed milk) was recommended after dosing, to increase drug absorption. Patients who vomited a dose within 1 h of treatment were given a full replacement dose, but no more than two doses were to be replaced during the three-day treatment period.

Antipyretics, such as paracetamol, were used to control high fever. In the event of severe malaria or danger signs, the patient was hospitalized and received intravenous quinine. Rescue therapy according to local treatment guidelines was also administered in cases of early or late treatment failure [23] or if a replacement dose was vomited within 2 h of administration.

### Evaluation

Follow-up visits took place on days 3, 7, 14, 28, and 42 after enrolment or at any time point whenever the child was sick. Patients who prematurely discontinued either study drug or the study were scheduled for a final visit before or on day 42. If they did not attend the day 42 visit, the final outcome of the malaria episode was determined where possible. Vital signs and body temperature were assessed at regular intervals during the study. A 12-lead electrocardiogram (ECG) was recorded at baseline and on day 3 (6-10 h after the last dose) and QTc calculated using Bazett's and Fridericia's QT correction formulae. Clinical signs and symptoms were assessed at baseline and before dosing, and any symptom that worsened or started after baseline was recorded as a new adverse event. An adverse event was defined as the appearance or worsening of any undesirable sign, symptom or medical condition occurring after starting the study drug even if the event was not considered to be related to study drug. An adverse event was considered serious if it was fatal or life-threatening, resulted in persistent or significant disability/incapacity or required inpatient hospitalization or prolongation of existing hospitalization. During the hospital stay and at every follow-up visit, adverse events were assessed for severity and association with study medication.

Thick and thin Giemsa-stained blood slides were prepared before each dose was administered and at every follow-up visit. Slides were examined by two independent microscopists and considered negative if no parasites were seen after examination of 200 oil-immersion fields in a thick blood film. Species determination was made based on assessment of thin films. Blood samples for PCR analysis were collected from every patient at baseline and at days 14, 28 and 42. PCR was performed centrally for all cases of recurrent parasitaemia after day 7 to distinguish recrudescence from reinfection.

### Pharmacokinetic assessment

The methodology has been described previously [24]. In brief, plasma concentrations of artemether and its major active metabolite dihydroartemisinin (DHA) were measured in the first 184 patients (two blood samples each), and lumefantrine concentration measured in the subsequent 625 patients (one blood sample each). For artemether and DHA, the two samples were taken at 1 and 2 h post first treatment dose (i.e. anticipated time of maximum concentration (C_max_)). C_max _was determined from these two concentrations, whichever was larger for a given patient. For determination of lumefantrine concentration, a sampling scheme was employed whereby samples were taken 6 h after dose 3 in 10% of patients, 6 h after dose 5 in 10% of patients, 6 h after dose 6 in 50% of patients (i.e. the expected time to C_max_), 24 h after dose 6 in 10% of patients, on day 7 in 10% of patients and on day 14 in 10% of patients. A population mean concentration-time curve was constructed by averaging all concentrations available for specified sampling intervals and taking the mean of the actual sampling times in each interval. C_max _and AUC_0-last _were derived from this population mean concentration-time curve.

### Study outcomes

The primary efficacy outcome was the 28-day PCR-corrected parasitological cure rate, defined as the proportion of patients with clearance of asexual parasitaemia within 7 days of initiation of study treatment, without recrudescence within 28 days and without use of rescue medication. Secondary outcomes included the 14-day and 42-day PCR-corrected parasitological cure rate, time to parasite, fever and gametocyte clearance, and safety and tolerability profiles.

### Statistical analysis

The intent-to-treat (ITT) population comprised all randomized patients with confirmed *P. falciparum *malaria who received ≥1 full dose of study drug and underwent ≥1 relevant post-baseline efficacy assessment while on treatment. The modified intent-to-treat (mITT) population consisted of all ITT patients who completed 28 days with a valid PCR assessment (if parasitaemia was present at day 28) or who were classified as treatment failures (i.e. discontinued study drug and received rescue treatment), but excluded patients who (i) received anti-malarials or anti-malarial antibiotics before day 28 for reasons other than rescue medication; (ii) had two replacement doses and vomited a subsequent dose within 1 h or vomited the replacement dose within 2 h; (iii) switched to rescue medication therapy during the three-day treatment period; or (iv) experienced a PCR-confirmed reinfection by day 28 or had missing/unclear PCR results at day 28. The safety population comprised all patients who received ≥1 dose of study medication and underwent at least one post-baseline safety assessment.

The primary end-point of 28-day PCR-corrected cure rate was assessed in the mITT population and presented with 95% confidence interval (CI) values. Time to parasite and fever clearance was evaluated by Kaplan-Meier estimates. Pharmacokinetic parameters are presented descriptively. For this evaluation, data from patients receiving the dispersible formulation of AL or standard crushed tablets were combined since the two formulations have been shown to be similar in terms of all measured efficacy, safety and pharmacokinetic parameters [21].

Data are presented according to the following weight groups: 5 to < 10 kg, 10 to < 15 kg (in order to further characterise potential differences in response between these two body weight groups despite the same dosing regimen), 15 to < 25 kg and 25 to < 35 kg.

## Results

### Patient population

A total of 899 patients were randomized, of whom 168 weighed 5 to < 10 kg, 379 weighed 10 to < 15 kg, 289 patients (32.1%) weighed 15 to < 25 kg, and 63 patients (7.0%) weighed 25 to < 35 kg. The ITT population included 886 patients (98.6%) and the mITT population included 812 patients (90.3%) (Table 1). The most frequent reason for exclusion from the mITT population was the absence of a day 28 parasite count in patients who had not been previously classified as a failure (n = 68). Overall, 782/899 patients (87.0%) completed the study. Baseline characteristics according to body weight group are summarized in Table 2. There were 15 patients (1.7%) aged less than 6 months (13 in the mITT population [1.6%]).

**Table 1:** Number of participants by study population

	Body weight				
	
Number of participants	All	5 to < 15 kg		15 to < 25 kg	25 to < 35 kg
		
		5 to < 10	10 to < 15		
Randomized population	547	168	379	289	63

ITT population^a^	535	164	371	288	63

mITT population^b^	477	143	334	277	58

Safety population	547	168	379	289	63

Patients could have more than one reason for exclusion from the ITT and mITT populations.

^a ^Reasons for exclusion from ITT population: no relevant post-baseline efficacy assessment (n = 12), no full dose of study medication taken (n = 9) and no *P. falciparum *at baseline (n = 1)

^b ^Reasons for exclusion from the mITT population were as for the ITT population, and additionally no day 28 parasite count without being a failure prior to day 28 (n = 68), vomiting of replacement dose or two replacements and subsequent vomiting (n = 16), reappearance of parasite but no valid PCR analysis until day 28 (n = 7), switch to rescue for other reasons (n = 4) and use of concomitant anti-malarials other than rescue medication (n = 4)

**Table 2:** Baseline characteristics (safety population)

Baseline Characteristics	Body weight			
	
	5 to < 10 kg (n = 168)	10 to < 15 kg (n = 379)	15 to < 25 kg (n = 289)	25 to < 35 kg (n = 63)
Body weight (kg)				
Mean(SD)	8.3 (1.1)	12.1 (1.4)	18.1 (2.6)	28.1 (3.0)
Median (range)	8.5 (5.0-9.9)	12.0 (10.0-14.7)	17.8 (15.0-24.5)	27.0 (25.0-34.0)

Female gender, n (%)	86 (51.2)	182 (48.0)	136 (47.1)	16 (25.4)

Age, months				
Mean (SD)	14.4 (6.7)	35.9 (13.5)	71.7 (22.4)	119.3 (20.5)
Median (range)	14 (0.0-42)^a^	35 (6-70)	71 (31-148)	122 (78-152)

Temperature,°C				
Mean (SD)	37.9 (1.2)	38.1 (1.1)	37.9 (1.0)	38.0 (1.0)

Parasite density, /μL				
Mean (SD)	49,571 (51,536)	53,921 (57,572)	40,974 (41,763)	21,850 (24,867)
Median (range)	31,937	35,400	26,640	13,040
	(0-228,000)	(520-628,571)	(2,039-192,610)	(1,581-109,720)

^a ^Start of treatment in one patient was recorded as day 21 after documented date of birth

### AL dosing and pharmacokinetics

In total, 866/899 patients (96.3%) completed the six-dose treatment regimen. One hundred and eighty-four patients provided two blood samples each for pharmacokinetic assessment of artemether and its major active metabolite DHA, and 625 patients provided one sample each for measurement of lumefantrine concentration. As reported elsewhere [24], there were no clinically relevant between-group differences in C_max _for artemether or DHA; lumefantrine C_max _and AUC _0-last _appeared higher in the 15 to < 25 kg group compared to the 5 to < 15 kg body weight group, approximately in line with the increase in dose, in particular for AUC after crushed tablet. In view of potential concerns about exposure in the smallest patients, a further pharmacokinetic sub-group analysis was performed for patients weighing 5 to < 10 kg and 10 to < 15 kg (Table 3). These patients all received the same regimen of one tablet twice daily for three days. Artemether and DHA C_max _were 2.15- to 3.4-fold higher in the 5 to < 10 kg body weight group compared to the 10 to < 15 kg body weight group. There were no apparent differences for lumefantrine C_max _or AUC between the two body weight groups.

**Table 3:** AL dosing and pharmacokinetic parameters in patients weighing 5 to < 10 kg or 10 to < 15 kg

	Body weight	
	
	5 to < 10 kg	10 to < 15 kg
**Artemether & dihydroartemisinin (DHA)**		

Dispersible tablet	N = 17^1^	N = 35^2^

Total dose artemether (mg/kg)	14.8 ± 2.57	10.0 ± 1.30

C_max _artemether (ng/mL)	372 ± 251	110 ± 97.7

C_max _DHA (ng/mL)	107 ± 76.6	38.2 ± 42.4

Crushed tablet	N = 18^3^	N = 37

Total dose artemether (mg/kg)	14.7 ± 1.94	10.0 ± 1.28

C_max _artemether (ng/mL)	295 ± 214	137 ± 111

C_max _DHA (ng/mL)	89.9 ± 83.5	36.6 ± 28.7

**Lumefantrine**		

Dispersible tablet	N = 24	N = 78

Total dose lumefantrine (mg/kg)	90.4 ± 9.46	60.4 ± 7.37

C_max _(μg/mL)	6.55	5.55

AUC_0-last _(μg·h/mL)	550	393

Crushed tablet	N = 26	N = 75

Total dose lumefantrine (mg/kg)	88.6 ± 13.1	59.5 ± 6.87

C_max _(μg/mL)	5.50	6.29

AUC_0-last _(μg·h/mL)	578	558

Data are mean ± SD;^1 ^N = 18 for DHA;^2 ^N = 34 for DHA;^3 ^N = 19 for DHA

### Age according to body weight dosing group

**Figure 1:**
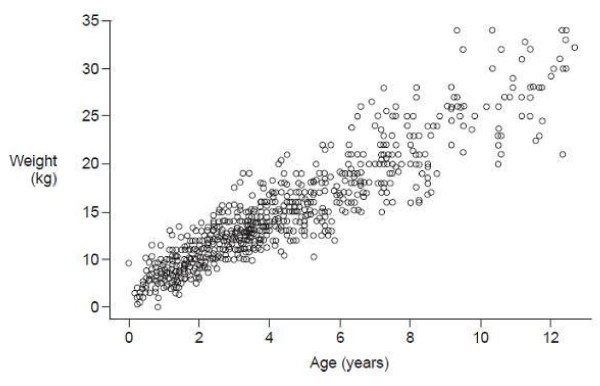
**Scatter plot of weight at baseline visit according to age (safety population)**.

Figure 1 illustrates the distribution of age and weight within the study population. As expected, there was a progressively greater distribution of weight for a given age in older children. Table 2 summarizes the mean age in each body weight group. Analysis of age by body weight showed that 80% (10-90th percentile) of patients weighing 5 to < 15 kg, 15 to < 25 kg and 25 to < 35 kg were aged 10-50 months, 46-100 months and 90-147 months, respectively.

### Efficacy

Each body weight group showed a 28-day PCR-corrected cure rate > 97% (5 to < 10 kg 97.2%, 10 to < 15 kg 98.8%, 15 to < 25 kg 97.8%, 25 to < 35 kg 98.3%). The 28-day PCR-corrected cure rate was comparable across all three body weight groups, with overlapping 95% CI values, i.e. there was no indication that outcome was affected by body weight group (Table 4). PCR cure rates at 14 days and 42 days were also similar in different body weight groups (Table 4).

**Table 4:** Efficacy outcomes by body weight group

	Body weight			
	
Efficacy outcomes	5 to < 10 kg	10 to < 15 kg	15 to < 25 kg	25 to < 35 kg
14-day PCR cure^a^				
n/N	140/143	334/334	277/277	58/58
%	97.9	100.0	100.0	100.0
95% CI (%)	94.0-99.6	97.9-100.0	98.7-100.0	90.8-100.0

28-day PCR cure^a^				
n/N	139/143	330/334	271/277	57/58
%	97.2%	98.8%	97.8%	98.3%
95% CI (%)	93.0-99.2%	97.0-99.7%	95.3-99.2%	90-8-100.0%

42-day PCR cure^a^				
n/N	123/129	284/290	227/237	50/53
%	95.3	97.9	95.8	94.3
95%	90.2-98.3	95.6-99.2	92.4-98.0	84.3-98.8

Kaplan Meier estimates of parasite clearance time, h^b^				
N	164	371	288	63
Median	35.6	35.0	25.8	25.8
Interquartile range	24.0-36.1	23.9-36.1	23.8-35.9	23.9-35.7
Mean (SE)	35.0 (1.2)	32.8 (1.0)	31.7 (1.1)	30.8 (1.6)

Kaplan Meier estimates of fever clearance time, h^b^				
N	164	369	288	63
Median	8.2	7.8	7.8	7.7
Interquartile range	7.7-35.0	7.5-23.6	7.6-23.4	7.4-8.5
Mean (SE)	44.4 (9.1)	26.5 (3.5)	30.5 (6.4)	11.5 (1.4)

^a ^mITT population^b ^ITT population

There was a tendency towards longer time to parasite and fever clearance in the lower body weight groups (Table 4). A meaningful assessment of gametocyte clearance according to body weight group was not possible due to the low number of patients with gametocytaemia at baseline (n = 41).

### Safety

The majority of adverse events were symptoms related to malaria (either the initial infection or the recurrent one). Across the total study population, the most frequent adverse events were pyrexia (36.9%), cough (24.2%), *P. falciparum *infection (20.8%) and vomiting (16.8%), the majority of which could be attributed to malaria. Including adverse events related to malaria, the cumulative rate of adverse events to day 42 was 74.4%, 69.9%, 67.8% and 61.9% in patients weighing 5 to < 10 kg, 10 to < 15 kg, 15 to < 25 kg and 25 to < 35 kg, respectively. There was a decreasing incidence of vomiting and pyrexia and an increasing incidence of headache and *P. falciparum *infection with higher body weight. The cumulative incidence of these adverse events to day 42 in the 5 to < 10 kg, 10 to < 15 kg, 15 to < 25 kg and 25 to < 35 kg cohorts was, respectively: vomiting, 32.1%, 16.4%, 11.4% and 3.2%; pyrexia, 42.3%, 38.3%, 34.9% and 23.8%; headache: 0.6%, 3.7%, 13.5% and 19.0%; *P. falciparum*: 16.7%, 20.1%, 23.2% and 25.4%. Other than vomiting (n = 75, 8.3%), no adverse event was reported to have a suspected relation to study drug in more than five patients. Serious adverse events were rare, occurring in 1.4% patients (n = 13) overall: 4.8% in the 5 to < 10 kg group, 0.8% in the 10 to < 15 kg group, 0.7% in the 15 to < 25 kg group and none in the 15 to < 25 kg group. In the 5 to < 10 kg group, serious adverse events (14 events in eight patients) included two episodes of initially severe malaria and two late malaria episodes on days 26 and 29 (one was a PCR-confirmed reinfection; one had no PCR data available). No serious adverse events were suspected to be related to study drug.

Study medication was discontinued due to adverse events in 20 patients (2.2%). Vomiting was the most frequent of these events (n = 17/22 adverse events). The most frequent reason for discontinuation of the study was adverse events (n = 70, 7.8%) and loss to follow-up (n = 27, 3.0%). The type of adverse event leading to study discontinuation was not recorded, but the protocol stipulated that any patient who required a second replacement dose of study drug was to be discontinued.

Three deaths occurred: one on day 31 due to a new and severe *P. falciparum *infection after clearing parasites within 24 h of the initial infection (5 to < 10 kg group), one on day 3 due to an unspecified infection accompanied by severe dehydration after discontinuing AL due to vomiting on day 2, at which point the patient was free of infection (5 to < 10 kg group) and one on day 7 due to haemorrhage after scarification by a witch doctor (10 to < 15 kg group). No death was suspected to be related to the study drug.

## Discussion

In this large population of African infants and children with uncomplicated falciparum malaria, the high efficacy of AL was confirmed across all body weight groups. The 28-day PCR-corrected cure rate, the primary endpoint, exceeded 97% in each body weight group with no indication that outcome differed between groups. These findings confirm that a fixed-dose strategy according to certain body weight groups (i.e. use of discrete dosing for a continuous weight variable) does not result in under-dosing with reduced efficacy or, alternatively, overdosing and risk of toxicity, at the extremes of each body weight window. Thus, the current body-weight dosing recommendations for AL appear appropriate. Specifically, dosing advice for the lowest body weight group (5 to < 15 kg) and, indeed, for the smallest patients within this category (5 to < 10 kg) is adequate, as confirmed by the efficacy and safety findings. Additionally, analysis of age by body weight group in this population of children from sub-Saharan African countries showed there to be only a minor overlap of age ranges between body weight groups based on the 10-90th percentiles.

In endemic areas, effective anti-malarial treatment is particularly critical in young children, who are in the process of acquiring partial immunity and present with high parasite densities and acute clinical episodes that can progress rapidly into severe, life-threatening malaria [2]. AL exposure was highest in the smallest children with body weight 5 to < 10 kg, but these results should be interpreted with caution due to the small sample size in this body weight group. Plasma levels of artemether, DHA and lumefantrine in all body weight groups (even in the smallest children, weighing 5 to < 10 kg) remained within the ranges reported in studies of adult patients [3,5,24,25]. Lumefantrine exposure was apparently unchanged in the smallest (5 to < 10 kg) children. It is reassuring that there was no trend to any age group having reduced exposure to artemether, DHA or lumefantrine. These pharmacokinetic analyses did not, however, undergo formal statistical comparisons, so conclusions must be tentative. The tendency towards longer time to parasite and fever clearance in the lower body weight groups may be partly explained by the lower immunity of younger children. Moreover differences in time to parasite and fever clearance between body weight groups need to be interpreted with caution since the measurements were scheduled to take place at 8, 24, 36, 48, and 60 h after the start of treatment, i.e. there was no continuous monitoring. This may have magnified differences between values since, for example, fever clearance at 50 h will only have been recorded at 60 h. Regardless of these methodological issues, however, it is important to be aware that in malaria-endemic areas, infants may be particularly vulnerable to slower clearance rates for parasitaemia. This is because they may be starting to acquire protective immunity, and as a result the likelihood of detecting resistant parasites may be higher in this age group. Delayed clearance times have been phenotypically linked to artemisinin resistance in the Thai-Cambodian border [26]. However, such cases may not necessarily represent a true emergence of parasite resistance to the drug because due to this lower immunity, infants can present with clinical evidence of malaria despite a relatively low parasite count i.e. fewer parasites may induce symptoms in infants than in older children. Nevertheless, the relevance of this finding needs to be reassessed to further clarify its potential impact. The current study excluded patients below 5 kg in weight, in accordance with WHO guidelines [2], since the safety and efficacy of ACT remains to be confirmed in this particular population. There were, however, 15 patients (1.7%) aged less than six months but weighing more than 5 kg included in the study.

Tolerability of AL was good, with no new safety concerns observed. The most frequently reported events were symptoms of malaria i.e. pyrexia, vomiting, cough and *P. falciparum *infection. The higher rate of vomiting and pyrexia in smaller children is expected, and in accordance with previous reports [27] it is possible that the increased incidence of headache in the higher body weight groups may have reflected the fact that smaller children are less likely to verbalize symptoms such as headache than the older patients. Cough tends to occur more frequently in young (< 5 years) patients with malaria [28] and the relatively high incidence reported here (24.2%) reflects the predominance of younger children in our population. *P. falciparum *infection reported as an adverse event most likely represents new infections, due to the long follow-up period, i.e. 42 days. The assessment of adverse events in cases of malaria, however, remains challenging because of the high background of malaria signs and symptoms [29]. Here, clinical assessment prior to the first dose of AL was performed in an attempt to make the adverse event reporting as robust as possible, with only those events which worsened or first occurred after the start of treatment being considered treatment-emergent adverse events. Nevertheless, although ACTs are universally regarded as very safe and tolerable drugs, the possibility that serious and non-serious adverse events are more frequently detected with the widespread use of the drug should always be considered.

The study methodology was robust. The population was large and the age distribution, in which approximately 60% of patients fell into the 5 to < 15 kg body weight group, reflects the fact that young children are the most vulnerable to malaria morbidity and mortality [30]. As specified in the protocol, artemether and lumefantrine plasma concentration was determined in separate subpopulations at different stages of the trial, but numbers were adequate for reliable estimations of pharmacokinetic parameters. The statistical methodology was sound, with the percentage of patients excluded from the mITT population (9.7%) being in the range of the pre-specified maximum of 10%.

## Conclusion

The efficacy of AL in treating uncomplicated falciparum malaria was similar across body weight dosing groups in infants and children, with no clinically relevant differences in safety or tolerability even when the lowest body weight group was further divided into patients weighing only 5 to < 10 kg. Analysis of age by body weight group in this population of children from sub-Saharan African countries indicates that the AL dosage scheme is appropriate even in the youngest children.

## Competing interests

Novartis Pharma AG funded the study and provided editorial support for the manuscript. QB has received speaker fees and payments from Novartis to attend meetings relating to this trial. VW, ON, GL and CK are employees of Novartis. DU is employed by Medicines for Malaria Venture (MMV). The other authors have no conflicts of interest to declare.

## Authors' contributions

All authors contributed to the design of the study and assisted with data interpretation. QB, CM, RG, SM, AN, JL, HM, AM, MB, PO, DU and BO coordinated the study and supervised the enrolment and follow-up of patients. VW provided biostatistical support and GL conducted the pharmacokinetic evaluation. ON and CK acted as medical advisors. All authors participated in the preparation of the manuscript and approved the final version.
